# A study of the effectiveness of the BOPPPS model combined with FC-CBL in the standardized training of general medicine residents

**DOI:** 10.3389/fpubh.2026.1856743

**Published:** 2026-08-21

**Authors:** Meixia Xiao, Yujian Gao, Yashu Wang, Ziwei Lei, Shengming Shi

**Affiliations:** Department of General Practice, First Affiliated Hospital of Huzhou Normal University, The First People's Hospital of Huzhou, Huzhou, China

**Keywords:** an innovative teaching model, BOPPPS model, CBL, flipped classroom, general practice residency training

## Abstract

**Background:**

The standardized training of general practitioners is a crucial component of postgraduate medical education and a strategic initiative in China’s healthcare workforce planning.

**Objective:**

To evaluate the effectiveness of an innovative teaching model-flipped classroom (FC) combined with case-based learning (CBL) under the BOPPPS (Bridge-in, Objective, Pre-assessment, Participatory Learning, Post-assessment, Summary) framework-in the standardized training of general practice residents.

**Methods:**

Thirty-eight resident trainees participating in general practice standardized training in the First People’s Hospital of Huzhou City were randomly assigned to the FC combined CBL (BOPPPS/FC-CBL) group under the BOPPPS model or the LBL group with traditional teaching. Theoretical tests, practical skills assessments, clinical thinking evaluations, and teaching satisfaction surveys were used to evaluate the teaching effects of the two groups.

**Results:**

The BOPPPS/FC-CBL group demonstrated significantly higher scores than the LBL group in theory tests (82.46 ± 8.34 vs. 76.82 ± 7.13, *p* = 0.03, *d* = 0.76), practical skills (101.46 ± 3.23 vs. 99.26 ± 1.62, *p* = 0.01, *d* = 1.02), and clinical case evaluations (89.73 ± 2.34 vs. 86.34 ± 1.28, *p* < 0.001, *d* = 1.80). More importantly, large to very large effect sizes were observed for teaching satisfaction (*d* = 1.48) and self-directed learning capacity (*d* = 1.62), indicating a substantively meaningful educational impact beyond statistical significance. All measures of learning engagement and competency enhancement were significantly higher in the intervention group (*p* < 0.05).

**Conclusion:**

The BOPPPS/FC-CBL group significantly enhances learning outcomes and satisfaction in general practice residency training, supporting its strategic scaling in China.

## 1 Introduction

General practitioners are doctors who provide medical treatment, health education, health promotion, and disease prevention to individuals and families and are the “gatekeepers” of primary health care. The most important reason why China’s overall health service system lags behind that of developed Western countries is the low proportion of general practitioners to the total number of doctors in China. Therefore, the training of general practitioners is a strategic national priority, and innovating training models to cultivate high-quality GPs is key to strengthening primary care and implementing the national health workforce plan (1). There are various types of teaching models applied to the standardized training of general practice residents in China. Most of these models are based on the traditional, lecture-centered transfer of theoretical knowledge, which fails to better develop the trainees’ independent learning ability, teamwork ability, and clinical thinking ability (2). The exploration and evaluation of effective training models, such as the one presented in this study, are essential to advancing GP education and supporting broader health system objectives. Given this strategic importance, it is essential to examine whether current training models are adequately preparing general practitioners for their roles.

The BOPPPS model is a novel instructional model that emphasizes student-centeredness and consists of six phases: Bridge-in, Objectives, Pretest, Participatory Learning, Post-test, and Summary (3). The BOPPPS model focuses on interaction and feedback, making it suitable for helping teachers carry out targeted and structured teaching activities, thus achieving better teaching results. In recent years, several studies have demonstrated that the BOPPPS model is more effective than traditional teaching in improving teamwork ability, self-learning ability, and clinical thinking ability, and results in higher teaching satisfaction in various disciplines, such as ophthalmology, thoracic surgery, and gynecology. However, the application of the BOPPPS model in general medical education has not been fully explored. One promising approach that addresses these limitations is the BOPPPS model.

The Flipped Classroom (FC) is a teaching model that turns the traditional teaching model upside down by transforming students from “passive listeners” to “active participants” (4). Teachers prepare instructional videos or materials through the online platform and send the materials to students in a uniform manner before they attend class. Students are allowed to study at home at their own pace and then work through queries with the teacher and classmates in the classroom (5). This approach flips through the in-class and out-of-class features, realigning the time in and out of the classroom and shifting the initiative of learning from the teacher to the student (6). A complementary approach that also promotes active learning is case-based learning (CBL).

Case-based learning (CBL) is a process by which teachers help students develop clinical thinking and enhance their ability to analyze and solve problems independently by guiding them through the analysis of typical cases (7). CBL has been widely used in medical education around the world (8). It is a student-centered teaching method that exposes students to real-world scenarios (9). This process can fully mobilize students’ motivation and can better solve the problem of insufficient integration of clinical practice and traditional teaching. Research has shown that CBL is more effective in improving students’ ability to solve real-world problems when compared to traditional instruction (10, 11).

Both BOPPPS and CBL-FC teaching methods are beneficial to student learning, and the effectiveness of the combined BOPPPS and CBL-FC teaching methods in the standardized training of general practitioners is still poorly known. Therefore, we combined the BOPPPS teaching model with CBL-FC in the standardized training of general practitioners. This randomized controlled study investigated 38 medical students to compare the performance of the BOPPPS/CBL-FC group with traditional teaching in theory tests, practical skills, and clinical case studies. The BOPPPS/CBL-FC group was compared with traditional teaching to assess the advantages of this teaching model and to provide a new strategy for the standardized training of general practitioners.

Although both BOPPPS and FC-CBL have been independently studied in various medical education contexts, three critical gaps remain. First, no study has integrated all three approaches (BOPPPS + FC + CBL) into a single pedagogical model. Second, the application of these methods in general practice residency training—a unique educational context that requires integration of knowledge, skills, and community-based reasoning—has not been systematically evaluated. Third, previous studies have largely focused on knowledge outcomes, with limited attention to non-cognitive competencies such as self-directed learning and teamwork, which are essential for general practitioners who often work in primary care teams with minimal specialist support. Our study aims to address these gaps.

## 2 Participants and methods

### 2.1 Study participants

**Figure 1:**
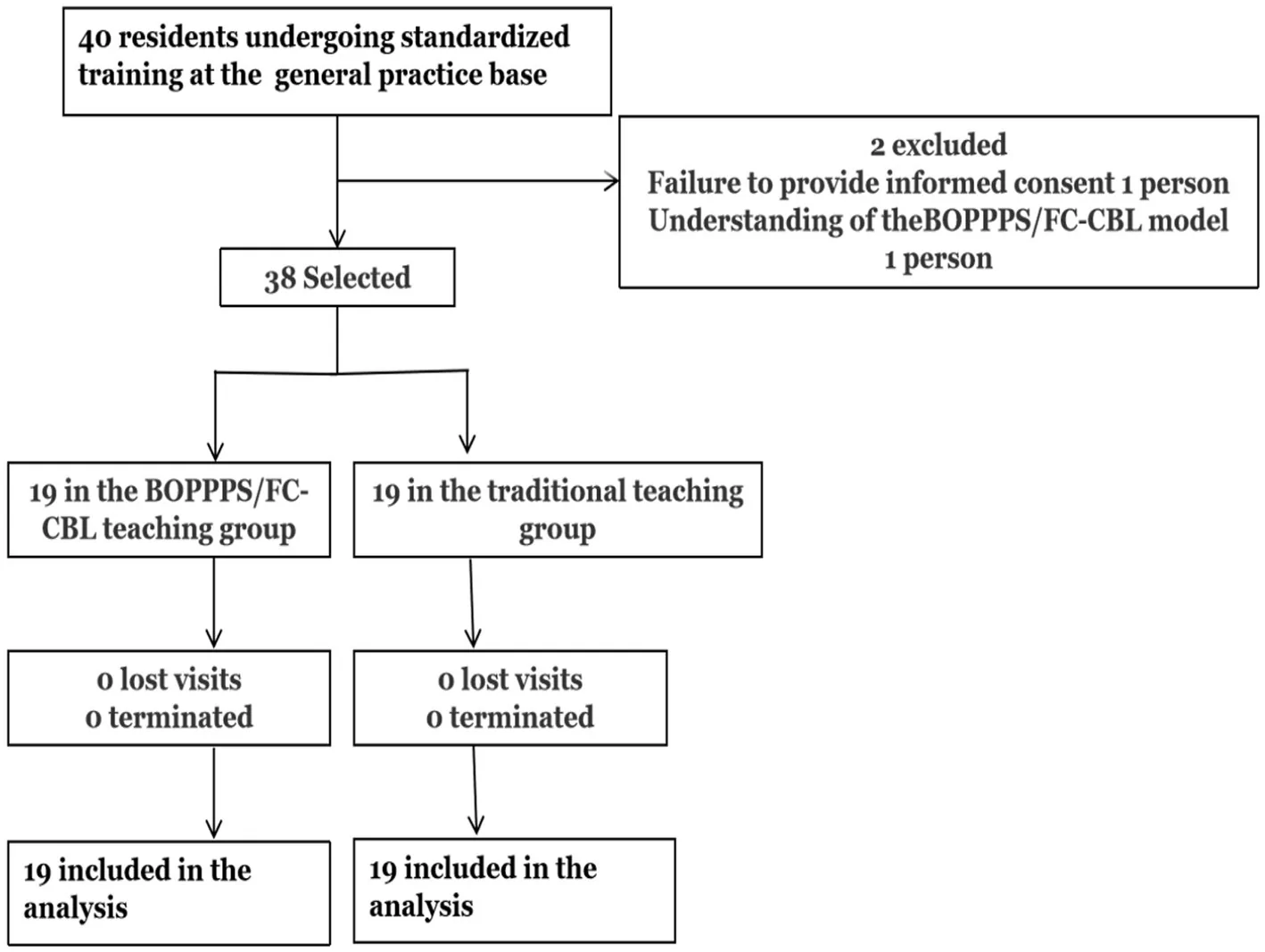
Flow chart of research subject enrollment.

A total of 40 participants were family medicine residents undergoing standardized training from January 2021 to December 2023 at our family medicine specialty site. The inclusion criteria were as follows: (1) Residents undergoing standardized training in general practice at our institution; (2) Voluntary participation in the study and signed informed consent; (3) Full-time clinical medicine undergraduate degree or higher; (4) No prior exposure to BOPPPS, flipped classroom, or FC-CBL teaching models; (5) Able to complete all teaching sessions, assessments, and questionnaires as required. The exclusion criteria were as follows: (1) Involuntary participation or withdrawal of informed consent; (2) Poor attendance or inability to complete the study procedures; (3) Leave of absence, rotation transfer, or other reasons leading to incomplete data. All responses were anonymized throughout the study. All methods were performed in accordance with the Declaration of Helsinki. After excluding residents who met excluded criteria, 95% (38 of 40) participated in the trial. Participants were randomly assigned to the BOPPPS combined with the FC-CBL (BOPPPS/FC-CBL) teaching group and the traditional lecture-based learning (LBL) group (see Figure 1). An independent statistician prepared a randomization table using SAS software (SAS Institute, Cary, NC, USA) and assigned study participants using simple randomization. Neither the participants nor the instructor knew the group information before the class. All participants were medical graduates from various medical colleges and universities across China, all holding a bachelor’s degree in clinical medicine and having passed the national medical licensing examination prior to enrollment in the residency program. The study was approved by the Institutional Ethics Committee of the First People’s Hospital of Huzhou City (No. 2024KYLL053-06). Written informed consent was obtained from all participants.

### 2.2 Research design

Both groups of trainees undergo a standardized 12-week residency training in the Department of Family Medicine. Both groups of trainees were tested on theoretical knowledge and practical skills before admission to the department, and the content of the tests was the same. The training instructors in both groups were attending general practitioners who had worked in the Department of Family Medicine of the First People’s Hospital of Huzhou City for at least 3 years. They were regularly trained as educators in BOPPPS/FC-CBL and LBL teaching methods and regularly passed the training according to the new standards for teacher training of general practitioners in Zhejiang Province. Both groups of trainees participated in teaching activities in the Department of Family Medicine, such as mini-lectures, difficult case discussions, and teaching rounds. The teaching content was based on the Chinese Residency Training in Family Medicine Syllabus.

### 2.3 Teaching implementation

The teaching implementation for the BOPPPS/FC-CBL group followed the structured six-phase model, as shown in Table 1. To provide a practical illustration, the community management of a patient with coronary heart disease (CHD) was used as a case example.

**Table 1:** Teaching implementation process of the BOPPPS/FC-CBL model.

Phase	Stage	Timing	Instructor activity	Learner activity	CHD case example
1	Bridge-in (B)	Before class	Creates an online group, shares a real case and guiding questions	Forms small groups (4–5 members), and begins the case discussion	“A middle-aged male presents with chest pain”—real-world scenario
2	Objectives (O)	Before class	Defines knowledge, skill, and affective objectives	Reads and understands learning objectives	Goal: Create a comprehensive community management plan for CHD
3	Pre-test (P)	Before class (day prior)	Posts online quiz using heuristic questions	Completes online quiz; results guide self-assessment	CHD definition, classification, diagnosis, and management basics
4	Participatory learning (P)	In class	Introduces the topic, facilitates discussion, and provides feedback	Group PowerPoint presentations; each learner expresses views; collaborative problem-solving	Groups present CHD case analysis; discuss long-term community management strategies
5	Post-test (P)	In class (end)	Conducts a quiz and a questionnaire to assess learning outcomes	Completes a quiz and a teaching effectiveness questionnaire	Short new CHD case to test application of learned principles
6	Summary (S)	After class	Leads knowledge summarization, recommends expert consensus, and clinical guidelines	Participates in summary discussion; reviews guidelines independently	Review CHD management guidelines; consolidate key learning points

#### 2.3.1 LBL group

Participants were encouraged to preview relevant textbooks, consensus statements, and guidelines before class. The instructor conducted traditional classroom instruction based on the syllabus, with the same content and objectives as the BOPPPS/FC-CBL group. At the end of the session, the teacher summarized the content and answered questions.

#### 2.3.2 Clinical rotations

Both groups of trainees participated in normal ward work according to the “General Practice Residency Training Syllabus,” including taking medical histories, physical examinations, writing medical records, and practicing clinical skills.

### 2.4 Effectiveness assessment

At the end of the trainees’ rotations in Family Medicine, both groups of trainees took the examination according to the Family Medicine Residency Training Syllabus, which included the theory test, practical skills, and clinical thinking in Family Medicine. The theory test paper was developed by the General Practice Teaching Secretary, with a score of 100 points, and was scrutinized by the Education and Training Department of the First People’s Hospital of Huzhou City. The practical skills exam focused on thoracentesis and was worth 110 marks. For the clinical thinking exam, which is worth 100 points, trainees draw lots to select a case for history taking, physical examination, diagnosis, differential diagnosis, and treatment. The clinical case used for the examination had not been previously used in teaching sessions for the BOPPPS/FC-CBL group. The clinical case used for the examination was identical between the two groups of trainees.

Both groups of trainees were required to complete an anonymous questionnaire (see Supplementary document) at the end of the training, which was used to assess the trainees’ evaluation of learning effectiveness and satisfaction. The questionnaire included learning efficiency, classroom atmosphere, interest in learning, level of knowledge acquisition, self-directed learning ability, teamwork skills, clinical thinking skills, and satisfaction with teaching. The questionnaire was assessed using a five-point scale, where a score of 1 indicated “very satisfied” and a score of 5 denoted “very dissatisfied,” with intermediate values representing “relatively satisfied,” “generally satisfied,” and “not very satisfied” (12).

### 2.5 Data evaluation and statistical analysis

The survey items of theoretical knowledge, practical skills, clinical thinking, and teaching satisfaction were statistically analyzed using SPSS 22.0 software. All measurements were expressed as (mean ± standard deviation). The data were tested for normality, and the test scores were consistent with a normal distribution, while the teaching satisfaction survey items were inconsistent with a normal distribution. Independent samples t-test was used for test scores, and the chi-square test was used to compare teaching satisfaction between the two groups. *p* < 0.05 was considered statistically significant and *p* > 0.05 was considered not significant.

As this study was a preliminary randomized controlled trial conducted in a single training center, the sample size was primarily determined by the number of eligible residents available during the study period (January 2021 to December 2023), rather than by a pre-study power calculation. However, a post-hoc power analysis was performed using G*Power 3.1 software for the primary outcome (theoretical test scores). With an effect size of 0.76 (calculated from the mean difference and pooled standard deviation), a sample size of 38 participants (19 per group), and an alpha level of 0.05, the achieved statistical power was 0.85, indicating sufficient power to detect significant differences in the primary outcome.

In order to go beyond mere *p*-value reporting and further assess the practical significance of the study findings, we calculated effect sizes. For quantitative data (exam scores), we used Cohen’s *d* to indicate the effect size; for non-parametric data (questionnaire scores), we calculated the effect size using *r* = Z/√*N* (where *Z* is the standardized statistic from the Mann–Whitney U test). For the primary outcome measure (theory test scores), a *post-hoc* power analysis was conducted using G*Power 3.1 software. Under the conditions of *α* = 0.05 and an observed effect size *d* = 0.76, the statistical power was 0.85, indicating that the current sample size is sufficient to detect significant differences between groups.

## 3 Results

### 3.1 Baseline comparison between the two groups

The BOPPPS/FC-CBL group consisted of 19 trainees (8 females, 11 males) with a mean age of (23.12 ± 0.32) years, mean theoretical score on admission (75.53 ± 1.12), mean practical skills score on admission (86.23 ± 1.23), and mean clinical case score on admission (84.21 ± 2.11). The LBL group consisted of 19 trainees (9 females and 10 males) with a mean age of (23.26 ± 0.19) years. The mean theoretical score on admission (75.26 ± 1.36), the mean practical skills score on admission (85.17 ± 2.32), and the mean clinical case score on admission (83.56 ± 1.23). The results showed that there was no significant difference between the baseline data of the two groups of trainees (*p*>0.05), and they were comparable, as shown in Table 2.

**Table 2:** Comparison of baseline data between the two groups of participants.

Groups	BOPPPS/FC-CBL group (*n* = 19)	LBL group (*n* = 19)	*χ^2^/t* values	*p* values
Sex, (Male/female)	8/11	9/10	0.10	0.75^a^
Average age	23.12 ± 0.32	23.26 ± 0.19	1.64	0.11^b^
Entry-level theory scores	75.53 ± 1.12	75.26 ± 1.36	0.67	0.51^b^
Admission to practice results	86.23 ± 1.23	85.17 ± 2.32	1.76	0.09^b^
Incoming clinical case grades	84.21 ± 2.11	83.56 ± 1.23	1.16	0.26^b^

BOPPPS/FC-CBL group, Combined teaching of BOPPPS and FC-CBL.

LBL group, Lecture-based instruction.

a The chi-square test was used to compare the two groups.

b A *t*-test was used to compare the two groups.

### 3.2 Comparative analysis of test scores

The mean score of the theory test in the BOPPPS/FC-CBL group was (82.46 ± 8.34), and the mean score of the theory test in the LBL group was (76.82 ± 7.13). The mean score of practical skills in the BOPPPS/FC-CBL group was (101.46 ± 3.23), and the mean score of practical skills in the LBL group was (99.26 ± 1.62). The mean score of clinical cases in the BOPPPS/FC-CBL group was (89.73 ± 2.34), and the mean score of clinical cases in the LBL group was (86.34 ± 1.28). The mean clinical case score was (89.73 ± 2.34) in the FC-CBL group and (86.34 ± 1.28) in the LBL group. The differences were statistically significant (*p* < 0.05), as shown in Table 3.

**Table 3:** Comparative analysis of test scores (mean ± standard deviation).

Groups	Theoretical results	Practical skills achievement	Clinical case scores
BOPPPS/FC-CB (*n* = 19)	82.46 ± 8.34	101.46 ± 3.23	89.73 ± 2.34
LBL (*n* = 19)	76.82 ± 7.13	99.26 ± 1.62	86.34 ± 1.28
*T* values	2.24	2.65	5.54
*p* values	0.03	0.01	*<* 0.001

BOPPPS/FC-CBL group, Combined teaching of BOPPPS and FC-CBL.

LBL group, Lecture-based instruction.

An independent samples *t*-test was used.

### 3.3 Comparison of teaching satisfaction and competency enhancement

As shown in Table 4, the BOPPPS/FC-CBL group scored significantly higher than the LBL group across all measures (*p* < 0.05). Critically, the effect sizes for self-directed learning capacity (Cohen’s *d* = 1.62) and teaching satisfaction (Cohen’s *d* = 1.48) were very large. Effect sizes of *d* > 1.5 are rare in educational research and suggest a fundamental shift in the learning process—from passive reception to active participation—rather than a marginal improvement in exam performance. In contrast, the LBL group showed small to negligible effect sizes for the same competencies, as shown in Table 4.

**Table 4:** Comparison of teaching satisfaction and competency enhancement between the two groups.

Dimension	Items	BOPPPS/FC-CBL group	LBL group	*p* values	*T* values	Effect size
Teaching experience	Teaching satisfaction	4.08 ± 0.34	3.24 ± 0.16	< 0.001	9.74	*d* = 1.48
	Classroom atmosphere	3.56 ± 0.75	3.12 ± 0.15	0.02	2.47	*d* = 0.80
	interest in learning	3.92 ± 0.12	3.18 ± 0.98	< 0.001	3.27	*r* = 0.47
Competency enhancement	Level of knowledge acquisition	4.12 ± 0.16	3.56 ± 0.35	< 0.001	6.34	*d* = 1.34
	Self-directed learning capacity	3.91 ± 0.15	3.15 ± 0.26	< 0.001	11.03	*d* = 1.62
	Teamwork skills	3.78 ± 0.12	2.98 ± 0.45	< 0.001	7.49	*d* = 1.18
	Clinical thinking skills	3.56 ± 0.49	2.82 ± 0.72	< 0.001	3.70	*d* = 0.96
	Learning efficiency	3.92 ± 0.45	3.23 ± 0.28	< 0.001	5.68	*d* = 0.94

The BOPPPS/FC-CBL group was taught using a hybrid teaching model integrating BOPPPS and FC-CBL, while the LBL group received lecture-based learning. Differences between the two groups were analyzed using independent-samples *t*-tests. All comparisons used the Mann–Whitney U test. Effect size: *d* = Cohen‘s, *r* = Z/√N.

## 4 Discussion

### 4.1 Summary of principal findings

The findings of this study are neither trivial nor uninteresting when examined through the lens of competency-based medical education. While the BOPPPS/FC-CBL group demonstrated significantly higher scores in theoretical tests, practical skills, and clinical case evaluations, the truly compelling contribution of this study lies in the large to very large effect sizes observed for key educational outcomes (13). Specifically, the effect size of *d* = 1.62 for self-directed learning capacity represents an educationally transformative impact—a level of improvement that is uncommon in medical education intervention studies. Similarly, the very large effect size for teaching satisfaction (*d* = 1.48) directly addresses the pervasive problem of learner burnout and disengagement in residency training. These findings indicate that this teaching model does not merely produce marginally better exam performance but fundamentally transforms how residents learn and perceive their training experience. Having established the overall effectiveness of the intervention, we now examine how these findings compare with existing literature.

### 4.2 Comparison with existing literature on BOPPPS in medical education

The effectiveness of the BOPPPS model has been documented across various medical disciplines, but its application in general practice residency training has remained largely unexplored. Our findings extend the existing literature in several important ways.

Previous studies have demonstrated the value of the BOPPPS model in discipline-specific medical education. For instance, Wang et al. (14) reported that a modified BOPPPS-based SPOC (Small Private Online Course) combined with a flipped classroom significantly improved oral histopathology learning outcomes among fifth-year undergraduates during the COVID-19 pandemic. Similarly, Li et al. (15) found that blended BOPPPS teaching enhanced learning outcomes and engagement in fermentation engineering courses. However, these studies were conducted in undergraduate or non-clinical settings, where learners are not simultaneously managing clinical responsibilities. General practice residency training presents a unique challenge: residents must integrate theoretical knowledge with hands-on clinical practice while working in primary care teams (16). Our study is the first to demonstrate that the BOPPPS framework can be successfully adapted to this demanding postgraduate training context.

Moreover, while previous BOPPPS studies have primarily focused on knowledge acquisition as the primary outcome, our study provides evidence that the BOPPPS framework, when combined with FC and CBL, can also develop non-cognitive competencies such as self-directed learning (*d* = 1.62) and teamwork skills (*d* = 1.18). This is particularly relevant for general practice education, where the ability to learn independently and collaborate effectively within primary care teams is essential for long-term professional development (17).

### 4.3 Comparison with existing literature on FC-CBL in medical education

The combination of flipped classroom and case-based learning has been investigated in several medical education contexts, and our findings are consistent with—and extend—this body of literature.

Yang et al. (8) demonstrated that FC-CBL was an effective teaching modality in nephrology clerkships, reporting significant improvements in academic performance and student satisfaction. Similarly, Cai et al. (9) found that FC-CBL improved undergraduate pathology education outcomes. Our study replicates these findings in the context of general practice residency training, with the BOPPPS/FC-CBL group showing significantly higher scores in theory tests (*d* = 0.76), practical skills (*d* = 1.02), and clinical case evaluations (*d* = 1.80) compared to the LBL group.

However, our study goes beyond previous FC-CBL research in three critical aspects. First, prior FC-CBL studies (8) did not embed their interventions within a structured pedagogical framework like BOPPPS. The BOPPPS framework provides explicit guidance for each stage of the learning process—from capturing initial interest (Bridge-in) to consolidating knowledge (Summary)—which likely enhanced the effectiveness of the FC-CBL approach (18). Second, previous studies focused primarily on knowledge outcomes, whereas we measured and reported effect sizes for competencies such as clinical thinking (*d* = 0.96) and self-directed learning (*d* = 1.62). Third, our study is situated in general practice residency training—a context that requires integration of knowledge, skills, and community-based reasoning—rather than in undergraduate or specialty-specific education (19). Beyond comparing our results with previous studies, it is important to understand why this combination works so effectively.

### 4.4 How the BOPPPS structure enhances learning

The BOPPPS framework’s six phases each contribute uniquely to the educational effectiveness observed in this study (20). The Bridge-in phase addresses the lack of contextual motivation in LBL by presenting real-world clinical dilemmas that immediately engage learners and demonstrate clinical relevance (21). The pre-test phase enables just-in-time teaching adjustments, allowing instructors to identify knowledge gaps before instruction begins—a feature entirely absent in traditional lectures, where all students receive identical content regardless of their baseline knowledge (22). The Participatory Learning phase, supported by FC and CBL, shifts learners from passive recipients to active problem-solvers. This active engagement has been shown in previous studies to improve long-term knowledge retention and clinical reasoning compared to passive listening (23, 24). Finally, the Post-test and Summary phases provide immediate feedback and knowledge consolidation, addressing the ‘forgetting curve’ phenomenon that often follows passive lecture-based instruction (25, 26).

### 4.5 Addressing the research gaps identified in the introduction

In the introduction, we identified three critical gaps in the literature. Our study directly addresses each of these gaps.

First gap: no previous study had integrated BOPPPS, FC, and CBL into a single pedagogical model. Our study provides the first evidence that these three approaches can be successfully integrated, with the BOPPPS framework serving as the structural backbone that organizes the FC and CBL components into a coherent learning sequence.

Second gap: the application of these methods in general practice residency training has not been systematically evaluated. Our study demonstrates that the BOPPPS/FC-CBL model is not only feasible but highly effective in this context, producing large to very large effect sizes across multiple competency domains. General practice education differs from specialty training in its emphasis on community-based, longitudinal, and holistic care—competencies that require integration of knowledge across multiple disciplines (27–29). Our findings suggest that the BOPPPS/FC-CBL model is well-suited to address these unique demands.

Third gap: previous studies have focused primarily on knowledge outcomes, with limited attention to non-cognitive competencies. Our study explicitly measured and reported effect sizes for self-directed learning (*d* = 1.62), teamwork skills (*d* = 1.18), and clinical thinking (*d* = 0.96)—competencies that are essential for general practitioners who often work in primary care teams with minimal specialist support. The large effect sizes observed for these competencies suggest that the BOPPPS/FC-CBL model may be particularly effective at developing the holistic competencies required for primary care practice.

### 4.6 Educational significance of the effect sizes

The effect sizes observed in this study warrant specific discussion, as they have important implications for medical education practice and policy. In educational research, effect sizes of d > 0.8 are generally considered large, while effect sizes of d > 1.2 are relatively rare and indicate substantial practical significance (23). Our finding that self-directed learning capacity showed an effect size of *d* = 1.62—well above this threshold—suggests that the BOPPPS/FC-CBL model produces an educationally transformative impact on this competency. Hattie’s (28) influential synthesis of over 800 meta-analyses in education found that the average effect size for educational interventions is approximately *d* = 0.40. By this benchmark, our observed effect sizes for self-directed learning (*d* = 1.62), teaching satisfaction (*d* = 1.48), teamwork skills (*d* = 1.18), and clinical thinking (*d* = 0.96) are exceptional. These large effect sizes suggest that adopting the BOPPPS/FC-CBL model could produce meaningful improvements in resident competencies within a relatively short time frame (12 weeks in our study). The question that naturally follows is why the BOPPPS/FC-CBL combination produces such large effect sizes.

### 4.7 Mechanisms: why the combination works

The synergistic effect of combining BOPPPS, FC, and CBL likely arises from the way these three approaches complement each other’s strengths and compensate for each other’s limitations.

The flipped classroom component addresses a key limitation of traditional CBL: the tendency for case discussions to be less productive when learners have not adequately prepared. By requiring pre-class preparation, FC ensures that all residents arrive at the Participatory Learning phase with a basic understanding of the relevant concepts, enabling more sophisticated case analysis during class time.

The BOPPPS framework addresses a limitation of standalone FC-CBL: the lack of explicit structure for each learning phase. While FC-CBL provides the core instructional strategies (pre-class preparation and in-class case discussion), it does not specify how to capture learner interest at the beginning (Bridge-in), how to provide structured feedback during learning (Post-test), or how to consolidate knowledge at the end (Summary) (30–32). The BOPPPS framework fills these gaps, creating a complete pedagogical cycle that guides both instructors and learners through each stage of the learning process.

Conversely, FC-CBL addresses a potential limitation of the BOPPPS model: the risk of becoming procedural without authentic content. The BOPPPS framework provides the structure, but FC-CBL provides the authentic clinical cases that make the structure meaningful. Without compelling cases to analyze, the Participatory Learning phase could become an abstract discussion of principles rather than an authentic problem-solving exercise (33).

Thus, the combination creates a whole that is greater than the sum of its parts: FC ensures preparation, BOPPPS provides structure, and CBL supplies authentic engagement. This synergistic effect likely explains the large to very large effect sizes observed across multiple outcome domains.

## 5 Implications and future directions

In conclusion, the BOPPPS/FC-CBL model represents an effective and satisfactory approach to the standardized training of general practice residents. By fostering clinical thinking, self-directed learning, and practical skills, this model aligns with the goals of modern medical education and supports the strategic development of a competent GP workforce in China. Widespread adoption and further evaluation of such innovative training methods are recommended to enhance the quality and scalability of GP training in line with national health workforce planning.

The BOPPPS framework‘s structured approach—from capturing initial interest (Bridge-in) to consolidating knowledge (Summary)—provides an effective organizational backbone that enhances the delivery of FC and CBL components. The flipped classroom ensures adequate preparation, enabling meaningful in-class engagement, while case-based learning provides authentic clinical contexts that motivate active problem-solving. Together, these three approaches create a synergistic effect that addresses the limitations of traditional lecture-based instruction, particularly its failure to develop self-directed learning and clinical reasoning skills.

These findings have significant implications for the strategic development of general practice education in China. Given the national priority to strengthen primary care through the training of competent general practitioners, the adoption of innovative teaching models such as BOPPPS/FC-CBL could contribute meaningfully to this goal. We recommend that medical education policymakers consider the strategic scaling of this model across training sites, while also investing in faculty development programs to ensure effective implementation.

## 6 Limitations and future research

However, the limitations of this study should be recognized. First, this study was conducted in only one hospital with a small sample size, and the generalizability of the results should be considered due to differences in educational settings and cultural backgrounds (34). Second, the FC-CBL group and the BOPPPS group were not included in this study, making it difficult to draw conclusions about which teaching methods work best in combination. Finally, the program in this study was maintained for only 12 weeks, making it impossible to assess the sustainability of this instructional model for teaching effectiveness. Fourth, the assessment of non-cognitive competencies relied on self-report questionnaires, which may be subject to social desirability bias. Future studies could incorporate objective measures of teamwork and clinical reasoning, such as observed structured clinical examinations (OSCEs) or peer evaluations, to provide more robust evidence. Fifth, the study did not examine the impact of learner characteristics—such as prior academic performance, learning style preferences, or motivation levels—on the effectiveness of the BOPPPS/FC-CBL model. Future research should explore these potential moderators to identify which types of learners benefit most from this approach. Therefore, rigorous large-sample randomized controlled center trials are needed to confirm our results and further explore the long-term effects of BOPPPS combined with FC-CBL learning.

## Data Availability

The raw data supporting the conclusions of this article will be made available by the authors, without undue reservation.
