# Supplementary material for: A study of the effectiveness of the BOPPPS model combined with FC-CBL in the standardized training of general medicine residents
Source: Front Public Health. 2026 Aug 21;14:1856743. doi: 10.3389/fpubh.2026.1856743 (PMC13541740; doi:10.3389/fpubh.2026.1856743)
Supplement: Supplementary file 1 [file Table_1.DOCX]

**Questionnaire**

**Survey on Learning Effect and Satisfaction of General Practice Trainees in the First affiliated Hospital of Huzhou University**

Dear general practice trainees,

Thank you very much for your participation in our evaluation of teaching methods. Please evaluate the methods used in this course. There are eight questions.The questionnaire was assessed using a FIVE-point scale, where a score of 5 indicated “very satisfied” and a score of 1 denoted “very dissatisfied”, with intermediate values representing “relatively satisfied”, “generally satisfied”, and “not very satisfied”.

1. How do you evaluate this teaching method in improving your Learning efficiency?

Your answer is	scores.

2.How do you evaluate this teaching method in improving your Classroom atmosphere?

Your answer is	scores.

3.How do you evaluate this teaching method in improving your interest in learning?

Your answer is	scores.

4.How do you evaluate this teaching method in improving your Level of knowledge acquisition?

Your answer is	scores.

5.How do you evaluate this teaching method in improving your Self-directed learning capacity?

Your answer is	scores.

6.How do you evaluate this teaching method in improving your Teamwork skills?

Your answer is	scores.

7.How do you evaluate this teaching method in improving yourClinical Thinking Skills?

Your answer is	scores.

8.How do you evaluate this teaching method in improving your Teaching satisfaction?

Your answer is	scores.
